# Co-Occurring Driver Genomic Alterations in Advanced Non-Small-Cell Lung Cancer (NSCLC): A Retrospective Analysis

**DOI:** 10.3390/jcm13154476

**Published:** 2024-07-31

**Authors:** Ilaria Attili, Riccardo Asnaghi, Davide Vacirca, Riccardo Adorisio, Alessandra Rappa, Alberto Ranghiero, Mariano Lombardi, Carla Corvaja, Valeria Fuorivia, Ambra Carnevale Schianca, Pamela Trillo Aliaga, Gianluca Spitaleri, Ester Del Signore, Juliana Guarize, Lorenzo Spaggiari, Elena Guerini-Rocco, Nicola Fusco, Filippo de Marinis, Antonio Passaro

**Affiliations:** 1 Division of Thoracic Oncology, European Institute of Oncology IRCCS, 20141 Milan, Italy; ilaria.attili@ieo.it (I.A.);; 2 Department of Oncology and Hemato-Oncology, University of Milan, 20141 Milan, Italy; 3 Division of Pathology, IEO, European Institute of Oncology IRCCS, 20141 Milan, Italy; 4 Division of Interventional Pulmonology, IEO, European Institute of Oncology IRCCS, 20141 Milan, Italy; 5 Department of Thoracic Surgery, IEO, European Institute of Oncology IRCCS, 20141 Milan, Italy

**Keywords:** molecular testing, next-generation sequencing, concurrent mutations, biopsy, tyrosine kinase inhibitors, treatments

## Abstract

**Background:** Actionable driver mutations account for 40–50% of NSCLC cases, and their identification clearly affects treatment choices and outcomes. Conversely, non-actionable mutations are genetic alterations that do not currently have established treatment implications. Among co-occurring alterations, the identification of concurrent actionable genomic alterations is a rare event, potentially impacting prognosis and treatment outcomes. **Methods:** We retrospectively evaluated the prevalence and patterns of concurrent driver genomic alterations in a large series of NSCLCs to investigate their association with clinicopathological characteristics, to assess the prognosis of patients whose tumor harbors concurrent alterations in the genes of interest and to explore their potential therapeutic implications. **Results:** Co-occurring driver alterations were identified in 26 out of 1520 patients with at least one gene alteration (1.7%). Within these cases, the incidence of concurrent actionable gene alterations was 39% (0.7% of the overall cohort). Among compound actionable gene mutations, *EGFR* was the most frequently involved gene (70%). The most frequent association was *EGFR* mutations with *ROS1* rearrangement. Front-line targeted treatments were the preferred approach in patients with compound actionable mutations, with dismal median PFS observed (6 months). **Conclusions:** Advances in genomic profiling technologies are facilitating the identification of concurrent mutations. In patients with concurrent actionable gene alterations, integrated molecular and clinical data should be used to guide treatment decisions, always considering rebiopsy at the moment of disease progression.

## 1. Introduction

Non-small-cell lung cancer (NSCLC) constitutes approximately 85% of all lung cancer cases, making it the most prevalent subtype worldwide [1]. The advent of precision medicine has illuminated the role of driver mutations in NSCLC pathogenesis. Within NSCLC, driver mutations play a significant role, with reported frequencies varying across populations, overall reaching up to 40–50% of NSCLC cases [2,3,4]. *EGFR*-activating mutations occur in about 10–15% of Caucasian and up to 50% of Asian NSCLC patients [5,6], while *ALK* and *ROS1* rearrangements are detected in 3–7% [7] and 2% of cases, respectively [8,9]. *KRAS* G12C mutations are overall the most frequent driver gene alteration (13%) [10]. Less common are *BRAF* V600 mutations (1–2%), *ERBB2* mutations (2–3%) and *RET* and *NTRK1*,*2*,*3* gene rearrangements (2% and 0.3%, respectively) [11,12,13,14]. These alterations are defined as actionable, representing key targets for tailored therapies, which can revolutionize treatment paradigms and improve patient outcomes. Indeed, tyrosine kinase inhibitors (TKIs) are currently the standard front-line treatment for *EGFR*-, *ALK*-, *ROS1*- and *BRAF*-positive patients, and targeted treatments are available in the pretreated setting or in clinical trials for *RET*, *ERBB2*, *KRAS* and *NTRK* actionable alterations [15].

On the other hand, non-actionable mutations are genetic alterations that do not currently have established targeted therapies or known treatment implications [16,17]. These mutations may still provide valuable information for understanding the biology of the tumor or predicting prognosis. Non-actionable mutations may include alterations in genes with unclear functional significance or those for which targeted therapies are still under investigation or development (i.e., *KRAS* non-G12C or *BRAF* non-V600 mutations). According to this definition, the use of comprehensive molecular profiling techniques, such as next-generation sequencing (NGS), may allow us to identify actionable and non-actionable mutations in most NSCLC samples [18].

Even more complex, the definition of concurrent mutations refers to the presence of multiple genomic alterations within the same tumor, often involving different driver genes [19]. These compound alterations can arise through various mechanisms, including clonal evolution, tumor heterogeneity and the selective pressure exerted by treatments [20,21,22]. The most frequent patterns of co-occurring alterations in NSCLC involve one actionable gene alteration and one alteration in genes involved in cell survival and proliferation (*TP53*, *STK11*, *PI3KCA* and DNA repair pathways), often defining a negative prognostic role compared with the non-co-mutated counterpart [18,19,23,24,25,26].

From a diagnostic standpoint, identifying and characterizing compound mutations requires comprehensive molecular profiling techniques. Additionally, interpreting the functional significance of these concurrent genomic alterations and predicting their impact on tumor behavior can be complex.

In clinical practice, the identification of concurrent actionable genomic alterations is a rare event (around 1%), described as anecdotical; however, these co-occurrences may influence treatment decisions [27,28,29]. Indeed, some combinations of mutations may confer resistance to specific targeted therapies, limiting their effectiveness. Conversely, certain compound mutations may offer opportunities for combination therapies targeting multiple pathways simultaneously, potentially enhancing treatment response. These aspects have been mainly investigated in the setting of acquired resistance to TKIs, but no data, besides case reports, are available for co-occurring actionable mutations present in treatment-naïve patients [30].

In the present study, we evaluate the prevalence and the pattern of concurrent driver genomic alterations in a large series of NSCLCs (i) to investigate their association with clinicopathological characteristics, (ii) to assess the prognosis of patients whose tumor harbors concurrent alterations in the gene of interest and (iii) to explore their potential therapeutic implications.

## 2. Materials and Methods

This retrospective analysis was conducted on a single institution’s database (European Institute of Oncology—Milan, Italy) consisting of 1520 patients diagnosed with advanced NSCLC harboring at least one driver mutation between December 2016 and December 2022. Patients diagnosed with advanced/metastatic NSCLC, molecularly profiled at our institution, were screened for the presence of co-occurrent alterations in genes of interest. Medical records were reviewed to collect information on molecular classification, demographic features, disease characteristics, treatments administered, responses and follow-up. Information on rebiopsy at disease progression, when performed, was also collected.

We considered genes of interest: *EGFR*, *KRAS*, *BRAF*, *ALK*, *ROS1*, *RET*, *MET*, *ERBB2*, *NTRK* and *PIK3CA*. The population of the current work was defined including patients with at least 2 co-occurring alterations in NSCLC driver genes (*EGFR*, *KRAS*, *BRAF*, *ALK*, *ROS1*, *RET*, *MET*, *ERBB2* and *NTRK*).

The identified co-occurrent alterations were then grouped into 3 categories based on their actionability. The first group consisted of patients with two actionable alterations, defined as *EGFR*-activating mutations, *KRAS* p.G12C mutations, *BRAF* p.V600 mutations, *ALK*, *ROS1*, *RET* and *NTRK* gene rearrangements, *MET* exon skipping mutations and *ERBB2* exon 20 mutations. The second group included patients with one actionable and one non-actionable alteration, while patients with two non-actionable mutations on driver genes were categorized into the third group (Supplementary Figure S1). Testing methods used for molecular classification included DNA and/or RNA NGS assays and FISH.

NGS analysis was performed using the Oncomine Comprehensive Assay (OCA) v.3 (Thermo Fisher Scientific, Waltham, MA, USA) according to manufacturer instructions. Libraries were prepared by using an Ion AmpliSeq DL8 kit (Thermo Fisher Scientific, MA, USA) on an Ion Chef system (Thermo Fisher Scientific, MA, USA) following manufacturer instructions. After library reamplification and barcoding, libraries were diluted at 30 pM and newly loaded into the Ion Chef instrument for automatic template preparation and chip loading. Finally, libraries were automatically loaded on an Ion 540™ Chip and sequenced on an Ion S5™ System (Thermo Fisher Scientific, MA, USA). Data analysis was performed as follows: after alignment to the hg19 human reference genome, coverage analysis with custom bed-files was assessed using the coverage plug-in (v.5.0.2.0) from Torrent Suite (v.5.0.2) (Thermo Fisher Scientific, MA, USA); the variant caller plug-in was used with a dedicated workflow on Ion Reporter Torrent Suite 5.16 (Thermo Fisher Scientific, MA, USA). Criteria for annotating and classifying molecular alterations as pathogenic/likely pathogenic according to gene mutation databases (Catalogue of Somatic Mutations in Cancer (COSMIC), cBioPortal for Cancer Genomics, ClinVar–NCBI–NIH) were a minimum coverage depth of 500×, allele coverage and a quality score ≥ 20 and a minimum variant allele frequency (VAF) of 5%.

FISH analyses were performed using a dual-color probe (ALK, ROS1 and RET IQFISH Break Apart Probe and MET IQFISH Probe with CEP7, respectively; Agilent Technologies, Santa Clara, CA, USA).

All the study procedures were carried out with general authorization for the processing of personal data for scientific research purposes from “The Italian Data Protection Authority” (http://www.garanteprivacy.it/web/guest/home/docweb/-/docwebdisplay/export/2485392, accessed on 10 April 2024). All information regarding patients was managed adopting anonymous numerical codes, and all samples were handled in compliance with the Helsinki Declaration.

Variables were presented by using the median value for continuous variables and percentages (numbers) for categorical variables.

Overall survival (OS) was defined as the time from the diagnosis of advanced/metastatic disease to death. Progression-free survival (PFS) was defined as the time from treatment start to disease progression or death. OS and PFS were estimated by using Kaplan–Meier methods. Median follow-up was calculated with the reverse Kaplan–Meier method. The Cox regression model was used for subgroup analysis on survival outcomes, and data were evaluated as hazard ratios (HRs) and their 95% confidence interval (CI), as appropriate. Given the very small subset and heterogeneity of patients, no adjustments were considered and only univariate analyses were performed.

The statistical significance level was set at *p* < 0.05 for all tests. All statistical analyses were performed with RStudio (RStudio: Integrated Development for R. RStudio, Inc., Boston, MA, USA, v.4.1.2).

## 3. Results

### 3.1. Frequency and Distribution of Compound Driver Gene Alterations

Overall, 1520 cases of advanced NSCLC with at least one alteration in genes of interest were identified in the study period. Co-occurring alterations in genes of interest were observed in 36 cases (2.3% of the patients with at least one actionable mutation). Among them, we identified 26 patients with at least two co-occurring driver genomic alterations, representing the population of interest in the current work.

The most frequent testing method was the combined use of DNA/RNA NGS plus FISH (53%), followed by DNA NGS plus FISH (41%) (Table 1).

**Figure 1:**
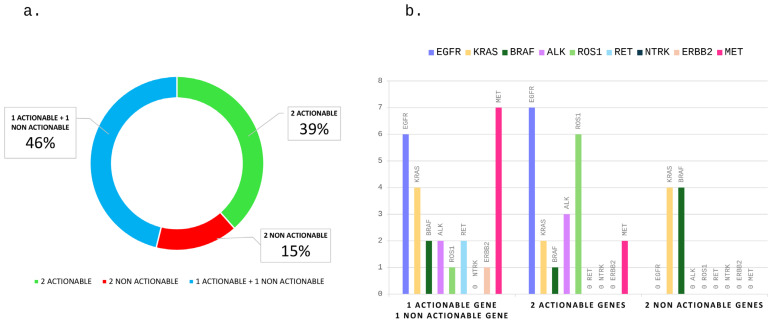
Distribution of concurrent driver genomic alterations. Distribution by actionability of compound driver genes (**a**) and distribution of compound mutations by involved genes (**b**).

The study population included *n* = 10 (39%) patients who had compound actionable alterations, *n* = 12 (46%) patients whose tumor harbored one actionable and at least one non-actionable alteration in driver genes and *n* = 4 (15%) patients with two or more non-actionable alterations in driver genes (Figure 1a; Table 2).

Overall, the most frequent alterations involved in the compound group are fusion genes (seven *ROS1* rearrangements and five *ALK* rearrangements), followed by *EGFR* mutations (*n* = 11). Among the ten cases with concurrent actionable alterations, nine harbored gene rearrangements and seven *EGFR* mutations. The most frequent association was an *EGFR* mutation with *ROS1* gene rearrangement (Figure 1b).

*KRAS* and *BRAF* gene mutations were the most frequent compound non-actionable mutations in driver genes (Figure 1b).

Of note, rebiopsy was performed at the time of disease progression in eight cases (50% of those with concurrent actionable mutations). In 87.5% of cases, the site of rebiopsy was different from that of the baseline, including one case of liquid biopsy. Considering the presence of one driver mutation, the molecular results of the rebiopsy analysis were concordant with those of the baseline analysis. However, loss of one gene alteration was observed in four (50%) cases, and in three cases, one additional resistant mutation was detected (Table 2).

### 3.2. Exploratory Evaluation of Therapeutic Approaches Used in Patients with Compound Actionable Gene Alterations

As expected, for patients with one actionable plus one non-actionable gene alteration, the therapeutic approach was chosen as per international guidelines.

**Figure 2:**
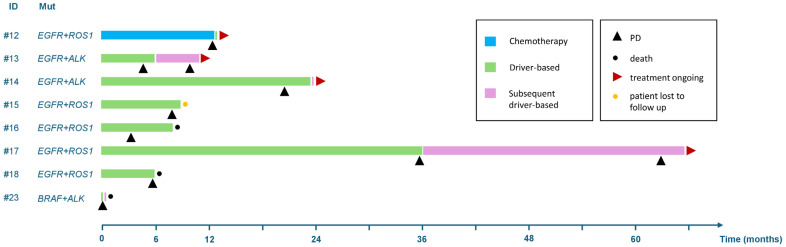
Swimmer plot of treatments in patients with co-occurring actionable gene alterations. The preferred treatment approach was the use of upfront targeted treatment (7 out of 8 patients), followed by an alternative driver-based targeted approach at disease progression in four cases. No subsequent treatment was possible in 2 patients due to worsening of clinical conditions and death.

In the group with compound actionable gene alterations (*n* = 10), the preferred treatment approach was an upfront targeted treatment (seven out of eight patients with available clinical follow-up), followed by an alternative driver-based targeted approach at disease progression in four cases. No subsequent treatment was possible in two patients due to worsening clinical conditions and death (Figure 2). The best responses to front-line targeted treatment were partial responses (4/7), stable disease (1/7) and progressive disease (2/7). Median PFS with front-line targeted treatment was 6 months (95% CI 3-NA), and median treatment duration was 8 months (95% CI 6-NA). Conversely, front-line chemotherapy was preferred only in one case, with 12-month lasting clinical benefit (Figure 2). Due to the unavailability of clinical trials in the setting, no patients were treated with dual TKIs.

### 3.3. Prognosis of Patients Harboring Compound Driver Gene Alterations

Follow-up data were available for eighteen out of twenty-six patients, including eight with concurrent actionable alterations, eight with compound actionable and non-actionable alterations and two in the concurrent non-actionable driver alteration group. The median follow-up was 28 months (IQR 22–70 months).

Overall, the median OS in the study population was 26 months (95% CI 22-NA).

Of note, the median OS was 25 months (95% CI 10-NA) in the compound actionable and 29 months (95% CI 22-NA) in the compound actionable plus non-actionable (HR 1.66, 95% CI 0.41–6.77) group.

In addition, no statistically significant differences in median survival were observed according to the presence or absence of the most represented genes.

## 4. Discussion

In NSCLC, concurrent molecular alterations pose both diagnostic and therapeutic challenges. We described a cohort of patients with NSCLC harboring concurrent alterations in driver genes (*n* = 26), including 10 patients with compound actionable alterations. We included in our study also *n* = 4 patients whose tumor harbored concurrent currently non-actionable *KRAS* and *BRAF* mutations, considering their potential future targetability with novel KRAS and BRAF inhibitors.

Overall, the incidence of co-occurring driver gene alterations in our cohort was 1.7%, with 50% of cases involving *EGFR* mutations. Within these cases, the incidence of concurrent actionable gene alterations was 39% (0.7% of the overall cohort). Among cases with compound actionable gene mutations, *EGFR* was the most frequently involved gene (70%).

Of note, a recently published retrospective study evaluated a total of 3077 patients with NSCLC who underwent molecular analysis by NGS, identifying 46 cases (1.5%) of co-occurring gene alterations, most (80%) involving EGFR mutations [28]. In this study, the incidence of compound driver actionable alterations was 41% (19 out of the 46 cases, 0.6%) [28]. However, the specific co-mutation patterns in this Asian population differed from our cohort. Indeed, in the Asian study, the most prevalent co-occurrence was represented by the association of *EGFR* and *ERBB2* actionable mutations (47%), whereas in our cohort, the most frequent association was *EGFR* mutations and *ROS1* gene rearrangements (50%). Of note, *ERBB2* actionable mutations were not observed in our compound study group, and *ROS1* rearrangements were not reported in the Asian compound population [28].

Due to the study period, testing methods were heterogeneous, and this might have represented a limitation of this study. Indeed, the time frame could affect the results due to the current modern molecular tools. First, we might have underestimated the real prevalence of co-occurring driver alterations; however, the incidence in our cohort was similar to those reported in the literature [28]. Second, we might have observed a higher rate of discordance at rebiopsy; however, this was only in one case, and different methods were used for baseline and progression analysis. Of note, the site of rebiopsy (progressive lesions) was different from that of the original diagnosis in all but one case, thus representing a good surrogate of tumor heterogeneity and clonal evolution under treatment-selective pressure.

Moreover, half of the rebiopsies were performed in patients with previously known co-occurring actionable mutations. Indeed, in the presence of concurrent actionable alterations, the need for molecular retesting at disease progression after any treatment is even stronger; this may allow for the identification of the persistence or disappearance of either clone, guiding subsequent treatment decisions [20].

In terms of treatment choice and outcomes, despite the very small numbers not powered to test for any difference nor to try valid estimations, we report a 6-month median PFS with front-line targeted treatments in patients with compound actionable mutations, which is quite low compared to the pivotal data of TKIs in *EGFR*-, *ALK*-, *ROS1*- and *BRAF*-mutated NSCLC patients. Of note, the population is highly heterogeneous (patients differ in age, driver mutation type and VAF), so these results are only exploratory. Another bias could indeed be due to the heterogeneity of the management protocols during the 6 years of the retrospective study.

In our opinion, the choice of a sequential targeted approach, which occurred in most (seven out of eight) patients, is a critical option. This choice might have been in part guided by the knowledge and treatments available at the time of treatment selection (the first approvals of front-line ALK TKIs and ROS1 TKIs in Italy occurred in 2017 and 2018, respectively). Such front-line choice may limit the considerations of the need for subsequent biopsy at disease progression because the focus was not on the presence of a dual driver. As such, assessing a subset of two patient cohorts before and after 2017 for ALK TKIs and before and after 2018 for ROS1 TKIs, respectively, could be an interesting continuation of this study.

Moreover, this approach does not consider the role of tumor heterogeneity and clone evolution during tumor progression. Therefore, we suggest a front-line agnostic treatment approach. Indeed, chemotherapy may exert cytotoxic effects on tumor cells and may be potentially effective across driver gene alterations, allowing the possibility to retest for actionable mutations to be targeted at disease progression. In our view, prospective clinical trials should be conducted to test this idea, not excluding the option of immunotherapy, looking at specific gene alterations and smoking status.

**Figure 3:**
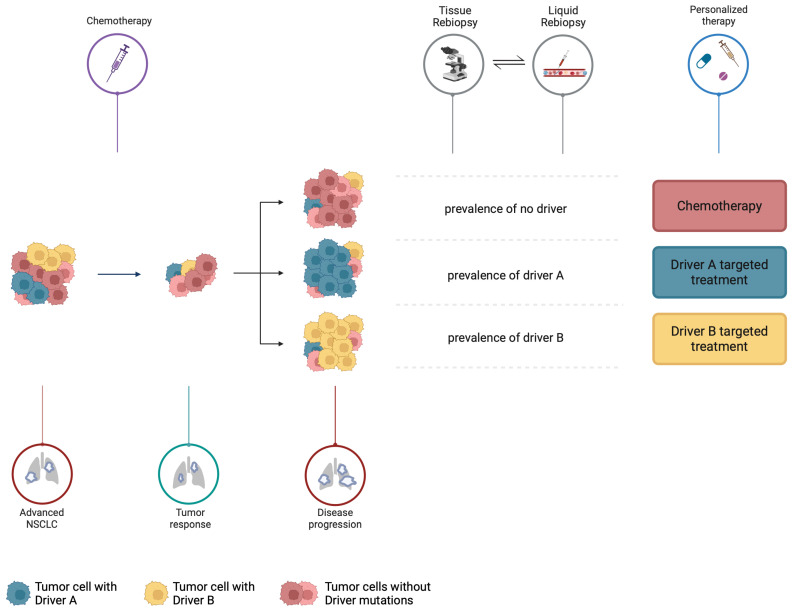
The proposed treatment approach to NSCLC with concurrent actionable genomic alterations. At diagnosis of advanced/metastatic NSCLC with compound driver actionable mutation, we propose front-line agnostic chemotherapy-based treatment. Indeed, chemotherapy exerts cytotoxic effects on tumor cells and is potentially active across driver gene alterations. At the time of disease progression, tumor rebiopsy, when feasible, should be performed to retest for residual driver mutations. To this end, the combined use of tissue rebiopsy and plasma liquid biopsy might investigate tumor heterogeneity more efficiently to detect the presence of residual or persistent mutations and their VAF, which might guide the choice of subsequent selected targeted treatments or subsequent agnostic approaches with chemotherapy-based regimens. Created with BioRender.com.

To this end, the combined use of tissue rebiopsy and plasma liquid biopsy might help to re-assess the presence of driver mutations and their VAF, which might guide the choice of subsequent selected targeted or agnostic treatment approaches (Figure 3).

## 5. Conclusions

Despite the above-discussed limitations of a small, retrospectively evaluated population, our exploratory results point out the clinical needs for the rare but not absent population of patients with co-occurring actionable gene alterations.

Overall, understanding the landscape of co-occurring genomic alterations in NSCLC is essential for optimizing treatment strategies and improving patient outcomes. Advances in genomic profiling technologies and computational tools for analyzing complex mutational profiles are facilitating the identification and interpretation of concurrent mutation, driving personalized approaches to cancer therapy.

## Figures and Tables

**Table 1:** Testing methods used for molecular assessment.

Testing Method	Baseline (*n* = 26)	Rebiopsy (*n* = 8)	Total (*n* = 34)
NGS DNA + FISH	12	2	14 (41%)
NGS DNA/RNA + FISH	14	4	18 (53%)
NGS DNA or DNA/RNA	0	1	1 (3%)
PCR alone	0	1	1 (3%)

**Table 2:** Clinicopathological characteristics and molecular alteration at baseline and progression in the study population (*n* = 26 patients with at least 2 co-occurring driver genomic alterations).

	Sex	Age ^1^	Smoking Status	Baseline			Progression			VAF ^§^			
Patient #				Baseline Biopsy Site	Collection Method	Baseline Testing Result	Rebiopsy Site (If Performed)	Collection Method	Rebiopsy Testing Result	Baseline Tumor Cellularity	Baseline VAF	Rebiopsy Tumor Cellularity	Rebiopsy VAF
**1**	M	68	never	RSL	TISSUE- BLOCK	*EGFR L858R* *KRAS Q61K*	-	-	-	40–60%	69% 7%	-	-
**2**	M	76	former	RSL LIL	CITO- BLOCK	*EGFR E906* *KRAS G12C*	-	-	-	60%	39% 50%	-	-
**3**	F	55	never	RPP LPP	CITO- BLOCK	*EGFR L858R* *ERBB2 S310F*	AL	TISSUE-BLOCK	*EGFR L858R* *ERBB2 wt*	20%	13% 9%	20–40%	6% -
**4**	F	78	never	RSL	TISSUE- BLOCK	*EGFR L858R* *MET L1213F*	RPP	TISSUE- BLOCK	*EGFR L858R* *MET wt*	60%	27% NA	40%	33% -
**5**	M	60	never	RSL	CITO- BLOCK	*EGFR L858R* *MET: c.2942-15_2942-12del*	RSL	TISSUE-BLOCK	*EGFR L858R* *MET: c.2942-15_2942-12del*	60%	57% NA	60%	62% NA
**6**	F	76	never	ML	CITO- BLOCK	*EGFR V843I* *ALK rearrangement (2p23)*	-	-	*-*	60%	5% NA	-	-
**7**	M	76	former	ML	CITO- BLOCK	*KRAS Q61H* *BRAF G464V*	-	-	*-*	40%	36% 23%	-	-
**8**	M	82	former	LIL	CITO- BLOCK	*KRAS K117N* *BRAF G469V*	-	-	*-*	20–40%	12% 12%	-	-
**9**	M	72	former	LIL	TISSUE- BLOCK	*KRAS G13D* *BRAF G469V*	-	-	*-*	60%	5% 17%	-	-
**10**	M	70	current	RIL	TISSUE- BLOCK	*KRAS Q61R* *BRAF D594G* *PIK3CA E453K*	-	-	*-*	20–40%	17% 10% 23%	-	-
**11**	F	52	never	RML	CITO- BLOCK	*KRAS G12V* *BRAF D594N* *RET KIF5B*	-	-	*-*	40%	11% 18% NA	-	-
**12**	M	62	never	LPP	TISSUE- BLOCK	*EGFR L861Q* *ROS1 rearrangement* *(6q22)*	LSL	TISSUE-BLOCK	*EGFR L861Q* *ROS1 rearrangement* *(6q22)*	20–40%	6% NA	40%	10% -
**13**	M	75	never	PLEURAL LIQUID	CITO- BLOCK	*EGFR L858R* *ALK rearrangement (2p23)*	RPP	TISSUE- BLOCK	*EGFR L858R* *EGFR T790M* *ALK rearrangement (2p23)*	-	.	20%	25% NA
**14**	F	71	never	T	TISSUE- BLOCK	*EFGR E746_T751delinsA* *EGFR amplification* *(30.2) chr7* *ALK rearrangement* *EML4 (ex 2)-ALK (ex 20*	-	-	*-*	40%	82% NA	-	-
**15**	F	62	current	T	TISSUE- BLOCK	*EGFR L858R* *ROS1 rearrangement* *(6q22)*	PLASMA TISSUE	LIQUID BIOPSY	*EGFR L858R* *EGFR T790M* *ROS1* not estimated *	20%	12% NA	NA	NA
**16**	F	84	never	RIL	CITO- BLOCK	*EGFR L858R* *ROS1 rearrangement* *(6q22)*	-	-	*-*	60%	95% NA	-	-
**17**	F	48	current	LPP	TISSUE- BLOCK	*EGFR E746_A750del* *ROS1 rearrangement* *(6q22)*	LINGULA	TISSUE- BLOCK	*EGFR E746_A750del* *EGFR T790M* *ROS1 wt*	20–40%	26% NA	40%	58% 32% NA
**18**	F	70	never	T	TISSUE- BLOCK	*EGFR L861Q* *ROS1 rearrangement* *(6q22)*	-	-	*-*	60%	40% NA	-	-
**19**	M	67	never	ML	CITO- BLOCK	*RET rearrangement* *(10q11)* *MET CNV (8.2) chr 7*	-	-	*-*	60%	NA NA	-	-
**20**	F	27	never	RPP	TISSUE- BLOCK	*ROS1 rearrangement* *(6q22)* *MET CNV (6.64) chr 7*	LPP	TISSUE-BLOCK	*ROS1 rearrangement* *(6q22)* *MET wt*	20–40%	NA NA	20%	NA NA
**21**	M	49	former	RML	CITO- BLOCK	*BRAF K601N* *MET ex14skipping*	-	-	*-*	60%	42% NA	-	-
**22**	F	59	former	RIL	TISSUE- BLOCK	*KRAS G12C* *ROS1 6q22* *PIK3CA E545K*	-	-	*-*	60%	7% NA 8%	-	-
**23**	F	69	current	RB	TISSUE- BLOCK	*BRAFV600E* *ALK rearrangement* *EML4 ex 19-ALK ex 20*	-	-	*-*	60%	18% NA	-	-
**24**	M	69	current	RSL	CITO- BLOCK	*KRAS CNV (6) chr12* *MET ex14skipping*	-	-	*-*	20–40%	NA 52%	-	-
**25**	M	68	former	RPP	TISSUE- BLOCK	*KRAS G12C* *MET ex14 skipping*	-	-	*-*	20%	10% 6%	-	-
**26**	F	40	never	ML	CITO- BLOCK	*ALK rearrangement* *EML4 ex 19-ALK ex 20* *MET CNV (23.87) chr 7*	-	-	*-*	60%	NA NA	-	-

**Abbreviations**: RSL = right superior lobe; RML = right middle lobe; RIL = right inferior lobe; LIL = left inferior lobe; LSL = left superior lobe; RPP = right parietal pleura; LPP = left parietal pleura; AL = axillar lymph node; ML = mediastinic lymph node; T = distal/carenal trachea; RB = right bronchus; NA = data not available § for concordant gene alterations. ^1^ Age at diagnosis of advanced/metastatic NSCLC. All patients were stage IV. * A different testing method from baseline was used in this case. # patient ID number.

## Data Availability

The raw data supporting the conclusions of this article will be made available by the authors on request.

## Supplementary Materials

The following supporting information can be downloaded at https://www.mdpi.com/article/10.3390/jcm13154476/s1. Figure S1 (Supplementary Figure S1): Study scheme. Diagram shows the study design and group categories defined.
