# Supplementary figures and images for: Co-Occurring Driver Genomic Alterations in Advanced Non-Small-Cell Lung Cancer (NSCLC): A Retrospective Analysis
Source: J Clin Med. 2024 Jul 31;13(15):4476. doi: 10.3390/jcm13154476 (PMC11313524; doi:10.3390/jcm13154476)

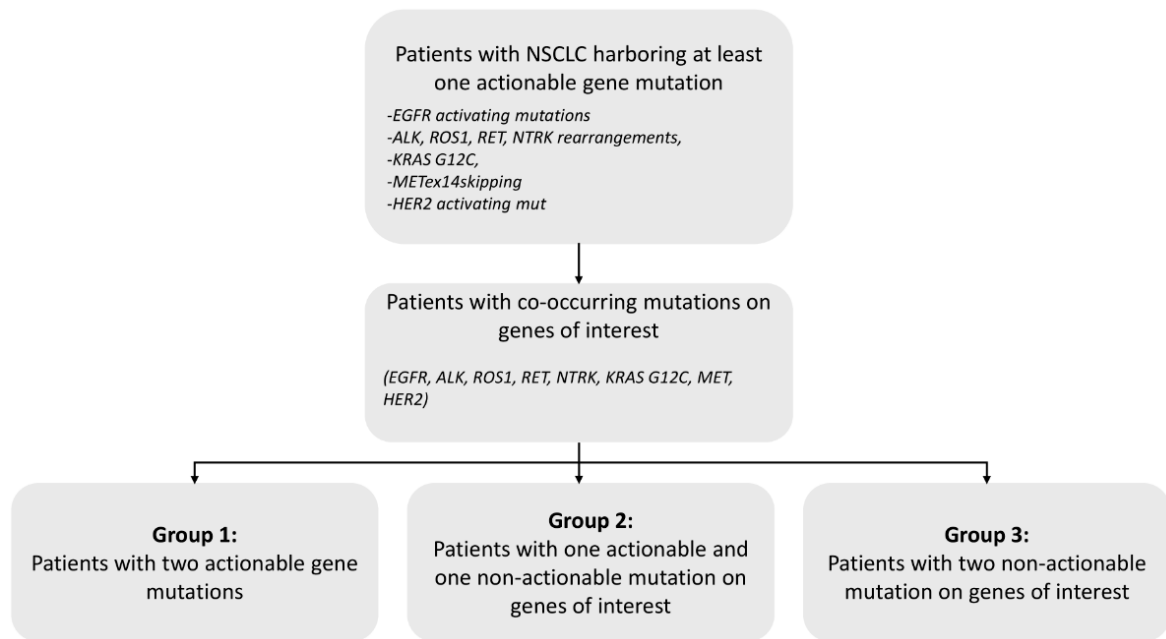

**Figure S1.** Study scheme. Diagram shows the study design and group categories defined.

Supplement: Supplementary file 1 [file jcm-13-04476-s001.zip › jcm-3055691-supplementary.pdf]
